# Burden of Respiratory Syncytial Virus Among Older Adults With an Acute Respiratory Infection: A Prospective Study in Six European Countries

**DOI:** 10.1093/cid/ciag146

**Published:** 2026-03-06

**Authors:** Manel Terns Riera, Rosa Prato, Alberto Pérez-Rubio, José María Echave-Sustaeta, Frederik Verelst, Shubhangi Gawade, Jean-Yves Pirçon, Pouya Saeedi, Philip Joosten, RSV-029 study group, Julio Ancochea Bermúdez, Laurence Bocqiua, Georg-Eike Böhme, Carlos Brotons Cuixart, Charles Bundy, Silvia Damaso, Nicolas Depaye, Cristina Genovese, Veronica Hulstrøm, Bastian Kirsch, Damien McNally, Helena Moza Moríñigo, Silvia Narejos Pérez, Carlo Pomari, Saul Robles, Fernando Sánchez Perales, Revathi Thimmaiah, Paul Torres Gutiérrez, Giacomo Tuana Franguel, Els Van de Paar, Claus Von Hessert

**Affiliations:** CAP el Remei, Vic, Spain; Hygiene Unit, Policlinico Foggia Hospital, University of Foggia, Foggia, Italy; Complejo Asistencial de Ávila, Ávila, Spain; Servicio de Neumologia, Hospital Universitario Quironsalud, Universidad Europea de Madrid, Madrid, Spain; GSK, Wavre, Belgium; GSK, Bangalore, India; GSK, Wavre, Belgium; GSK, Wavre, Belgium; GSK, Wavre, Belgium

**Keywords:** acute respiratory infection, community-dwelling older adults, prevalence, respiratory syncytial virus

## Abstract

**Background:**

Respiratory syncytial virus (RSV) primarily affects the respiratory tract, with a high burden among older adults. We aimed to estimate the prevalence and characteristics of RSV-associated respiratory infections in community-dwelling older adults across Europe.

**Methods:**

A prospective, observational study was conducted in six European countries over three consecutive RSV seasons (October 2021 until April 2024). Non-RSV vaccinated participants (age ≥60 years) presenting with symptoms of acute respiratory infection (ARI) at general practitioners and outpatient clinics/outpatient hospitals were recruited. Respiratory syncytial virus and other respiratory viruses were detected using a combined nasal and throat swab. Diary cards and regular phone contacts were used to collect information on onset and resolution of symptoms. The prevalence of RT-PCR confirmed RSV-ARI (cRSV-ARI) was estimated, as well as symptom duration, underlying comorbidities, prevalence of RT-PCR confirmed RSV lower respiratory tract disease (cRSV-LRTD), complications, hospitalizations, and co-infections.

**Results:**

A total of 2573 participants were enrolled (139 with cRSV-ARI). The prevalence of cRSV-ARI varied by season: 3.6% in Season 1, 9.3% in Season 2, and 4.2% in Season 3. The most frequently reported upper respiratory, lower respiratory, and systemic symptoms were nasal congestion/rhinorrhea, cough, and fatigue (cRSV-ARI: 83.1%, 97.8%, and 64.0%; non-cRSV-ARI: 78.0%, 95.0%, and 68.2%). The overall prevalence of cRSV-LRTD was 3.9% in Season 1, 12.5% in Season 2, and 7.3% in Season 3.

**Conclusions:**

This study highlights the substantial burden of RSV among older adults in Europe and reflects the shift in RSV epidemiology due to the COVID-19 pandemic, indicating a return to prepandemic seasonality trends.

**Clinical Trial Registration:**

Not applicable

## Teaser

Respiratory syncytial virus (RSV) presents a substantial burden in community-dwelling older adults in Europe. RSV seasonality appears to return to prepandemic levels.

**(See the Editorial Commentary by Branche on pages e1289–91.)**

Respiratory syncytial virus (RSV) is an RNA virus with two major antigenically distinct subtypes (RSV-A and RSV-B), both causing respiratory illness [1]. In temperate regions, infections with RSV mostly occur during the winter period, while in humid, tropical regions infections are more variable throughout the year [2, 3]. RSV is highly contagious and usually results in an acute respiratory infection (ARI) which resolves within 1–2 weeks. The main symptoms include mild to moderate nasal congestion, low grade fever, wheezing, and coughing [3]. In older or immunocompromised adults, complications of RSV infection can include pneumonia, bronchiolitis, as well as exacerbations of chronic obstructive pulmonary disease (COPD), congestive heart failure, or asthma, ultimately leading to respiratory failure and death in some cases [3].

RSV infections occur at all ages, with the highest burden and most complications occurring among infants, older adults (≥60 years of age), and individuals with underlying medical conditions (such as chronic heart or lung conditions) that increase the risk of a severe RSV episode [3, 4]. As the global population ages, the morbidity and mortality due to respiratory infections, including RSV, are rising among older adults [4–6]. Although the true prevalence of RSV amongst adults is likely underestimated due to a lack of testing, underascertainment, and underreporting [7, 8], recent reviews have highlighted the important burden of RSV in adults over 60 years of age [9, 10]. It is estimated that more than 3 million RSV cases, 270 000 hospitalizations and 19 500 in-hospital deaths due to RSV occurred in adults over 60 years of age in Europe in 2019 [10]. An additional hurdle in diagnosing RSV-associated ARI is the similarity of symptoms of RSV and influenza-like illness [11]. The contribution of respiratory viruses other than influenza in ARI is not well characterized in older adults.

To highlight the public health need and the potential benefit of recently approved vaccines against RSV, it is crucial to raise awareness of the burden of RSV among health care providers, patients, and policy makers. The aim of this study was to estimate the prevalence and characteristics of RSV-associated respiratory infections in Europe, in community-dwelling adults aged ≥60 years presenting with ARI. The impact on health-related quality of life, symptomology, health-care resource utilization, and days missed from work among study participants is reported in the accompanying publication [12].

**Figure 1.**
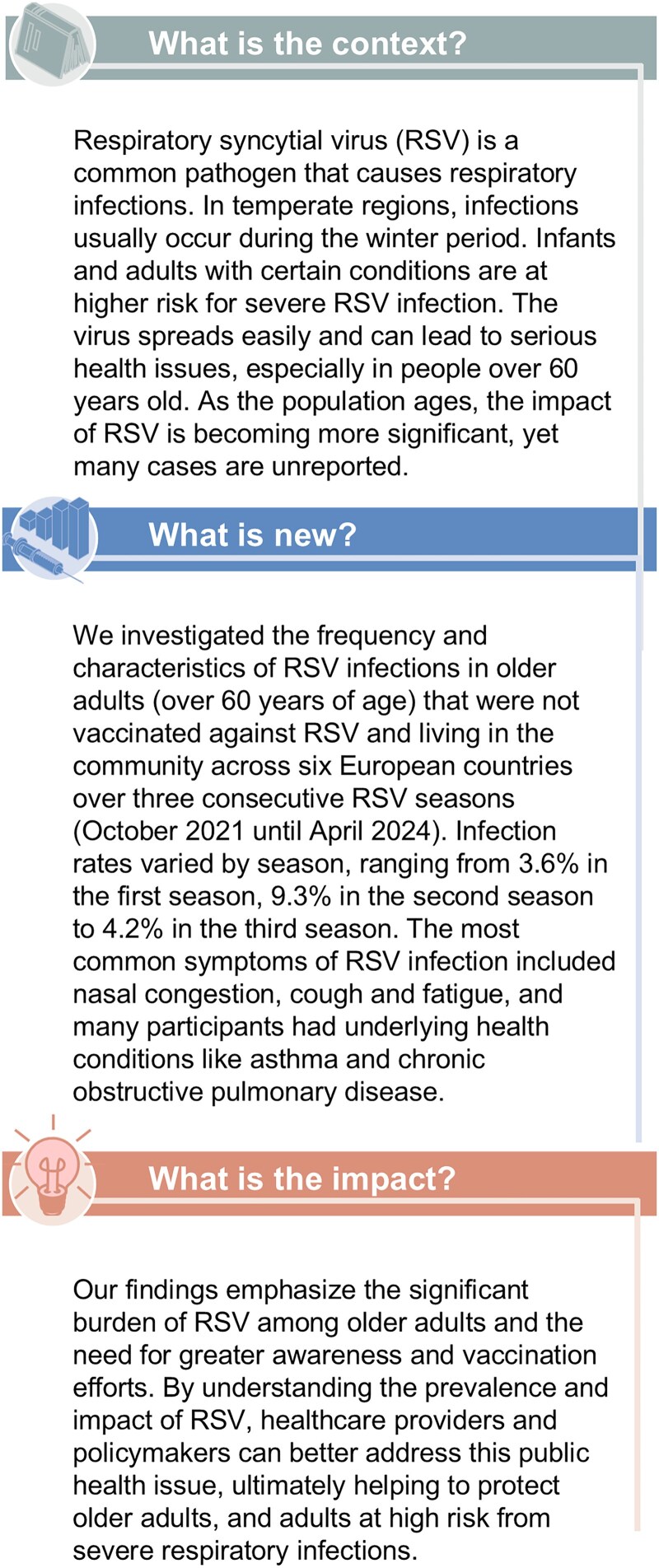
Plain language summary.

A summary of the study findings in plain language is presented in Figure 1.

## METHODS

### Study Design

This study was a prospective, observational study, conducted in six European countries (Finland, Germany, Italy, Poland, Spain, and the United Kingdom), between 01 October 2021 and 30 April 2024, thus including three consecutive RSV seasons and two post-season periods. Study centers were general practitioners’ (GPs) offices or outpatient clinics/hospitals.

The study was conducted in accordance with the ethical principles derived from international guidelines, including the Declaration of Helsinki and Council for International Organizations of Medical Sciences International Ethical Guidelines, as well as other applicable regulations. The study protocol and its amendments were approved by the relevant ethical review boards and informed consent was obtained from each participant before enrollment into the study.

### Study Population

Participants were recruited from patients ≥60 years of age seeking medical advice due to an ARI. Eligible patients had to exhibit at least two respiratory signs or symptoms of ARI (see Supplementary case definitions and Table 1 in the supplementary material) with onset less than 7 days prior to the enrollment visit. This time window was expanded to less than 10 days at the start of Season 2 (01 October 2022). The main exclusion criteria were a previous vaccination with any RSV vaccine and any planned administration of RSV-specific drugs or vaccines during the study period. Re-enrollment of participants, in the same RSV season or in subsequent RSV seasons, was permitted provided that the participant had at least a 7-day period free of ARI signs or symptoms, and the new ARI occurred after follow-up of the previous ARI (Day 29, or Day 57 if the ARI was not resolved at Day 29). A full list of inclusion and exclusion criteria can be found in the Supplementary material.

### Objectives

The primary study objective was to estimate the prevalence of reverse transcription polymerase chain reaction (RT-PCR) confirmed RSV-ARI (cRSV-ARI) among the participants, by season, country, age group, setting (GP or outpatient clinic/hospital), and RSV subtype. The secondary objectives included describing the duration and symptoms or signs of cRSV-ARI, describing the underlying comorbidities of participants with cRSV, estimating the prevalence of RT-PCR confirmed RSV lower respiratory tract disease (cRSV-LRTD), describing complications, hospitalizations, and number of deaths associated with cRSV, and estimating the prevalence of coinfection with other respiratory viruses (a full description of study objectives and endpoints can be found in Supplementary Table 2).

### Variables

Demographic data, medical history, and clinical signs or symptoms of ARI (with start and end date) were collected at Visit 1. A brief physical examination was conducted, and a combined nasal and throat swab was collected for the detection of RSV and other respiratory viruses by RT-PCR. Each participant was provided an ARI symptoms and medication diary card to collect information on the onset and resolution dates, as well as the medication(s) used.

A follow-up contact by phone was scheduled on Days 8, 15, and 29 (±3 days) after Visit 1 to assess the evolution and resolution of clinical signs or symptoms of ARI and potential complications, and medications used for ARI treatment. If the ARI was not resolved by Day 29, follow-up was extended with an additional phone contact on Day 57 after Visit 1. At study conclusion (Day 29 or Day 57), the investigator reviewed all the data collected and assessed any possible complication reported. Participants were instructed to contact the investigator in case of symptoms they perceived as serious. Any serious adverse event, judged by the investigator to be related to study procedures, was followed until study completion (Day 29 if ARI resolved or Day 57 if ARI not resolved by Day 29).

### Statistical Methods

The following “Season” definitions were applied: RSV Season, from 01 October to 30 April of the following calendar year; post-RSV Season, from 01 May to 30 September. A “Year” was defined as an RSV season and the following post-Season period (eg, Year 1 from 01 October 2021 to 30 September 2022). Since the study ran for two full years and one RSV season, we refer to the study period as Year 1, Year 2, and Season 3. The analysis set included all participants for whom RT-PCR results were available, without reported protocol violations leading to exclusion.

Sample size calculations assumed that an RSV infection would be detected by RT-PCR in 2% to 10% of participants with ARI per year [6, 13]. A sample size of 1500 for Year 1 and Year 2 would result in acceptable ranges around a prevalence point estimate of 4% with sufficient power to meet the study objectives. For Season 3, a sample size of 1000 was chosen.

All statistics were calculated descriptively, without multiplicity adjustments. Two-sided 95% confidence intervals (CIs) were calculated using the extended Clopper–Pearson exact CI for clustered data [14]. Participants that were enrolled more than once were considered independent in the analysis.

## RESULTS

### Study Participants

A total of 2779 participants were enrolled in the study (Year 1: 691; Year 2: 1083; Season 3: 1005, Figure 2). In Year 1, 18 individuals were enrolled two times, none of whom had cRSV-ARI; in Year 2, 23 participants were enrolled twice (two with cRSV-ARI), four participants were enrolled three times (none with cRSV-ARI), and three participants were enrolled four times (two with cRSV-ARI); during Season 3, 30 individuals were enrolled two times (five with cRSV-ARI). Thirteen participants were enrolled in all three study periods (Year 1, Year 2, and Season 3). Overall, 2573 distinct individuals participated in the study, of whom 139 had cRSV-ARI.

**Figure 2.**
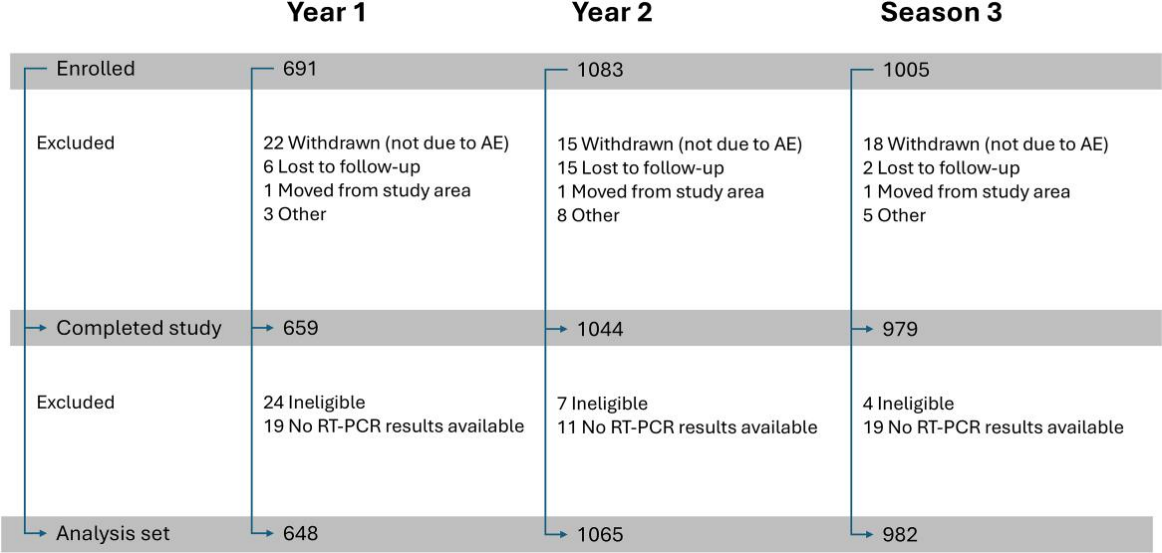
Participant disposition. Season 1, 01 October 2021 to 30 April 2022; Post-season 1, 01 May 2022 to 30 September 2022; Year 1, Season 1 and post-season 1; Season 2, 01 October 2022 to 30 April 2023; Post-season 2, 01 May 2023 to 30 September 2023; Year 2, Season 2 and post-season 2; Season 3, 01 October 2023 to 30 April 2024. In Finland, recruitment ended at the end of Season 1 (30 April 2022). In Germany and Poland, recruitment started at the beginning of Season 2 (01 October 2022). Abbreviations: AE, adverse event; RT-PCR, reverse transcription polymerase chain reaction.

Overall participant age at ARI onset ranged from 60 to 99 years, with a mean age of 68.7 ± 6.9 years during Year 1, 69.9 ± 7.3 years during Year 2, and 70.0 ± 7.2 years during Season 3 (Table 1). Most participants were women (Year 1: 57.7%, Year 2: 60.2%, Season 3: 62.1%) and of White-Caucasian ethnicity (Year 1: 97.2%, Year 2: 98.6%, Season 3: 99.4%). Demographic characteristics were balanced among study groups and periods.

**Table 1.** Participant Demographics (Analysis Sets)

	Year 1			Year 2			Season 3		
	cRSV-ARI	Non-cRSV-ARI	Total	cRSV-ARI	Non-cRSV-ARI	Total	cRSV-ARI	Non-cRSV-ARI	Total
N	19	629	648	76	989	1065	41	941	982
Age^a^: mean ± SD	67.8 ± 7.3	68.7 ± 6.9	68.7 ± 6.9	71.2 ± 7.7	69.8 ± 7.3	69.9 ± 7.3	68.8 ± 7.3	70.1 ± 7.2	70.0 ± 7.2
Age group^a^: n, %									
60–74	15, 78.9%	511, 81.2%	526, 81.2%	55, 72.4%	771, 78.0%	826, 77.6%	32, 78.0%	696, 74.0%	728, 74.1%
≥75	4, 21.1%	118, 18.8%	122, 18.8%	21, 27.6%	218, 22.0%	239, 22.4%	9, 22.0%	245, 26.0%	254, 25.9%
Sex: n (%)									
Female	11, 57.9%	363, 57.7%	374, 57.7%	49, 64.5%	592, 59.9%	641, 60.2%	27, 65.9%	583, 62.0%	610, 62.1%
Male	8, 42.1%	266, 42.3%	274, 42.3%	27, 35.5%	397, 40.1%	424, 39.8%	14, 34.1%	358, 38.0%	372, 37.9%
Country^b^: n (%)									
Finland	3, 15.8%	26, 4.1%	29, 4.5%	0	0	0	0	0	0
Germany	0	0	0	10, 13.2%	47, 4.8%	57, 5.4%	1, 2.4%	76, 8.1%	77, 7.8%
Italy	2, 10.5%	91, 14.5%	93, 14.4%	13, 17.1%	114, 11.5%	127, 11.9%	2, 4.9%	128, 13.6%	130, 13.2%
Poland	0	0	0	25, 32.9%	332, 33.6%	357, 33.5%	14, 34.1%	363, 38.6%	377, 38.4%
Spain	10, 52.6%	373, 59.3%	383, 59.1%	20, 26.3%	351, 35.5%	371, 34.8%	19, 46.3%	310, 32.9%	329, 33.5%
United Kingdom	4, 21.1%	139, 22.1%	143, 22.1%	8, 10.5%	145, 14.7%	153, 14.4%	5, 12.2%	64, 6.8%	69, 7.0%
Race: n (%)									
White-Caucasian	19, 100%	611, 97.1%	630, 97.2%	74, 97.4%	976, 98.7%	1050, 98.6%	41, 100%	935, 99.4%	976, 99.4%
Other	0	18, 2.9%	18, 2.8%	2, 2.6%	13, 1.3%	15, 1.4%	0	6, 0.6%	6, 0.6%

For two participants (with non-cRSV-ARI), the onset of first respiratory symptom occurred before the protocol-specified time window. These participants should have been excluded from the analysis; however, the error was discovered after database lock. No reanalysis was performed, as it only represents two out of 2573 distinct participants, and it is unlikely to have a meaningful impact on the results.

Year 1: 01 October 2021 to 30 September 2022; Year 2: 01 October 2022 to 30 September 2023; RSV Season 3: 01 October 2023 to 30 April 2024.

Abbreviations: ARI, acute respiratory infection; cRSV-ARI, confirmed RSV-ARI; n, number of participants in given category; N, number of participants with available results; RSV, respiratory syncytial virus; RT-PCR, reverse transcription polymerase chain reaction; SD, standard deviation.

^a^Expressed in years, at ARI onset.

^b^In Finland, recruitment ended at the end of Season 1. In Germany and Poland, recruitment started at the beginning of Season 2.

### Prevalence of RSV-ARI

**Figure 3.**
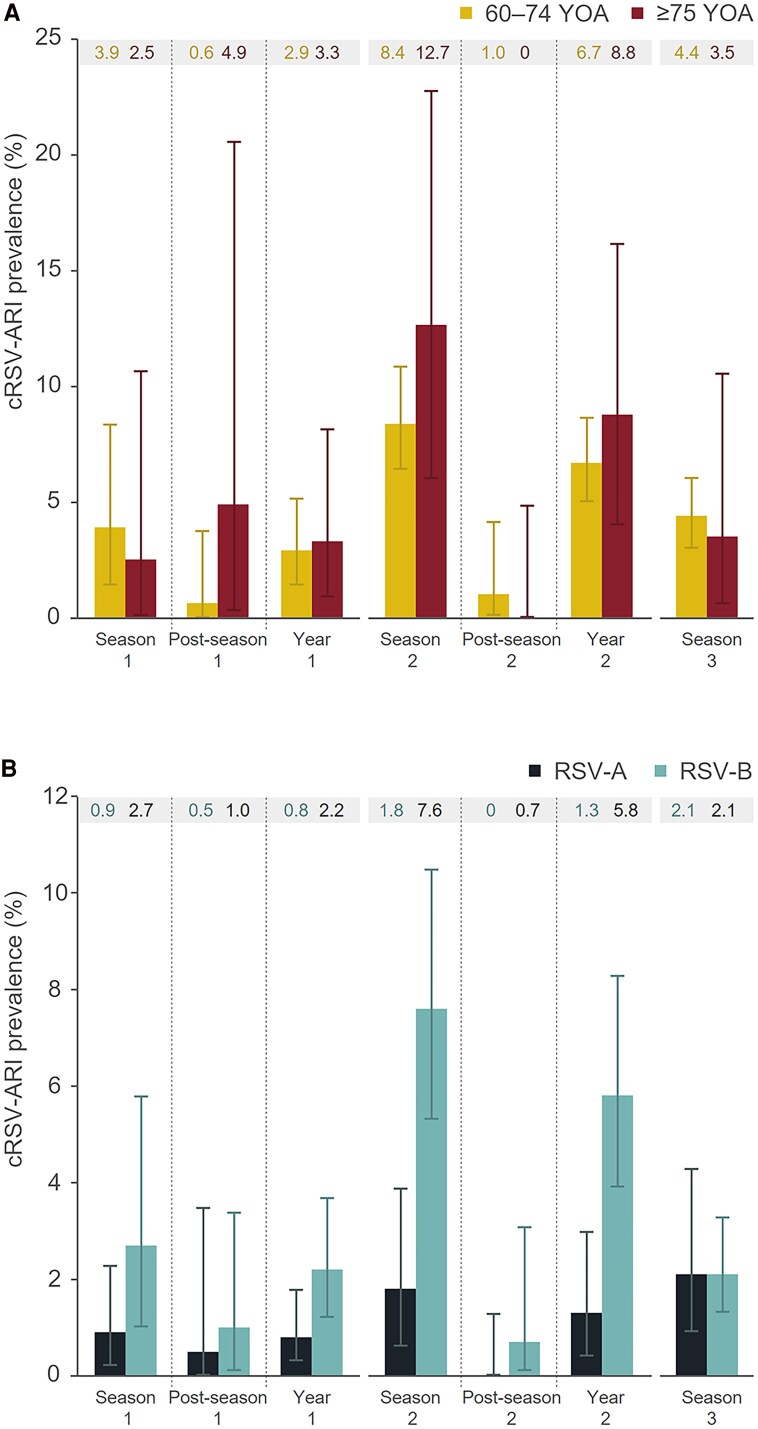
Prevalence estimates of RT-PCR confirmed RSV-ARI (analysis set) by age category (*A*) and RSV subtype (*B*). Numbers on shaded background are the point estimates. Further details are shown in Supplementary Table 3. Season 1, 01 October 2021 to 30 April 2022; Post-season 1, 01 May 2022 to 30 September 2022; Year 1, Season 1 and post-season 1; Season 2, 01 October 2022 to 30 April 2023; Post-season 2, 01 May 2023 to 30 September 2023; Year 2, Season 2 and post-season 2; Season 3, 01 October 2023 to 30 April 2024. Abbreviations: ARI, acute respiratory infection; cRSV-ARI, confirmed RSV-ARI; RSV, respiratory syncytial virus; RT-PCR, reverse transcription polymerase chain reaction; YOA, years of age.

During the first year, overall cRSV-ARI prevalence was 2.9% (95% CI: 1.5–5.0), with most episodes (16/19) occurring during the RSV season (Season 1 cRSV-ARI prevalence: 3.6% [1.4–7.5]), and mostly (14/19) caused by RSV-B (Year 1 RSV-B prevalence 2.2% [1.2–3.7]) (Figure 3; Supplementary Table 3). During Year 2, the overall prevalence was higher compared to Year 1 (7.1% [5.3–9.4]) mostly due to an increased prevalence during Season 2 (9.3% [7.2–11.8]). Post-season 2, the prevalence was comparable to post-Season 1. Similarly to Year 1, most cRSV-ARI episodes (62/76) were caused by RSV-B (prevalence: 5.8% [3.9–8.3]). During Season 3, the prevalence decreased to 4.2% (2.7–6.1), with an equal number of cRSV-ARI episodes caused by RSV-A and RSV-B (21/41 each, one participant tested positive for both subtypes, RSV-A prevalence 2.1% [0.9–4.3], RSV-B prevalence 2.1% [1.3–3.3]). Prevalence by country and setting are shown in Supplementary Table 4.

### Occurrence and Duration of Symptoms or Signs

Among participants with cRSV-ARI, upper respiratory symptoms, mainly nasal congestion/rhinorrhea, were reported by 91.9% (95% CI: 83.4–96.9) of participants, and lower respiratory symptoms or signs, mainly cough, were reported by 99.3% (96.0–100) of participants (Table 2). Among participants with non-cRSV-ARI, upper respiratory symptoms were reported by 91.4% (86.9–94.8), and lower respiratory symptoms or signs by 97.3% (96.6–97.9) of participants. Systemic symptoms or signs, mainly fatigue, were reported by 87.5% (76.7–94.5) of participants with cRSV-ARI, and 88.2% (79.7–94.1) of participants with non-cRSV-ARI. The symptoms or signs which occurred more frequently in participants with cRSV-ARI when compared to non-cRSV-ARI (difference >10%) were sputum production (72.8% [54.4–86.9] vs 61.6% [47.4–74.5]) and dyspnea (59.6% [50.7–68.0] vs 41.0% [33.0–49.3]).

**Table 2.** Occurrence and Duration of Symptoms and Signs of Acute Respiratory Infection, Combined Years 1, 2 and Season 3 (Analysis Set)

	cRSV-ARI			Non-cRSV-ARI			Total		
	n/N	% (95% CI)	Duration (days)	n/N	% (95% CI)	Duration (Days)	n/N	% (95% CI)	Duration (days)
Upper respiratory symptoms	125/136	91.9 (83.4–96.9)	…	2326/2544	91.4 (86.9–94.8)	…	2451/2680	91.5 (87.0–94.8)	…
Nasal congestion/rhinorrhea	113/136	83.1 (71.9–91.2)	16 (3–57)	1982/2541	78.0 (68.7–85.6)	14 (2–382)	2095/2677	78.3 (69.1–85.8)	14 (2–382)
Sore throat	89/136	65.4 (52.4–77.0)	10 (3–48)	1737/2541	68.4 (63.5–72.9)	10 (1–63)	1826/2677	68.2 (63.2–72.9)	10 (1–63)
Lower respiratory symptoms/signs	135/136	99.3 (96.0–100)	…	2477/2545	97.3 (96.6–97.9)	…	2612/2681	97.4 (96.7–98.0)	…
Cough^a^	133/136	97.8 (91.8–99.8)	19 (3–58)	2416/2543	95.0 (93.8–96.1)	16 (1–66)	2549/2679	95.1 (93.9–96.2)	16 (1–66)
Sputum production^a^	99/136	72.8 (54.4–86.9)	19 (5–52)	1563/2537	61.6 (47.4–74.5)	16 (1–68)	1662/2673	62.2 (47.8–75.1)	17 (1–68)
Dyspnea or shortness of breath^a^	81/136	59.6 (50.7–68.0)	14 (1–58)	1041/2539	41.0 (33.0–49.3)	12 (1–63)	1122/2675	41.9 (34.1–50.1)	12 (1–63)
Wheezing^a^	28/134	20.9 (13.6–29.9)	2 (1–14)	320/2529	12.7 (8.3–18.1)	12 (1–60)	348/2663	13.1 (8.7–18.6)	11 (1–60)
Crackles (rales)/rhonchi^a^	28/125	22.4 (13.1–34.2)	…	335/2466	13.6 (5.6–26.1)	…	363/2591	14.0 (6.0–26.3)	…
Respiratory rate ≥20 breaths per minute	11/127	8.7 (3.4–17.4)	…	183/2509	7.3 (3.9–12.3)	…	194/2636	7.4 (3.9–12.4)	…
Low or decreased oxygen saturation	9/129	7.0 (3.2–12.8)	…	160/2526	6.3 (4.1–9.2)	…	169/2655	6.4 (4.1–9.3)	…
Need for oxygen supplementation	1/135	0.7 (0.0–4.5)	…	26/2526	1.0 (0.5–1.9)	…	27/2661	1.0 (0.5–1.9)	…
Only upper respiratory symptoms	1/136	0.7 (0.0–4.0)	…	68/2544	2.7 (2.1–3.4)	…	69/2680	2.6 (2.0–3.3)	…
Only lower respiratory symptoms/signs	11/136	8.1 (3.1–16.6)	…	218/2544	8.6 (5.2–13.1)	…	229/2680	8.5 (5.2–13.0)	…
Both upper respiratory symptoms and lower respiratory symptoms/signs	124/136	91.2 (82.6–96.4)	…	2258/2544	88.8 (84.2–92.4)	…	2382/2680	88.9 (84.4–92.4)	…
Systemic symptoms/signs	119/136	87.5 (76.7–94.5)	…	2243/2542	88.2 (79.7–94.1)	…	2362/2678	88.2 (79.8–94.0)	…
Myalgia	64/136	47.1 (36.7–57.7)	12 (1–51)	1144/2537	45.1 (35.9–54.5)	11 (1–377)	1208/2673	45.2 (36.2–54.4)	11 (1–377)
Arthralgia	45/136	33.1 (25.2–41.7)	13.5 (1–56)	880/2537	34.7 (27.2–42.8)	11 (1–375)	925/2673	34.6 (27.2–42.6)	11 (1–375)
Fatigue	87/136	64.0 (46.4–79.2)	17 (1–62)	1734/2541	68.2 (57.8–77.5)	14 (1–63)	1821/2677	68.0 (57.4–77.4)	14 (1–63)
Headache	70/136	51.5 (42.8–60.1)	11 (1–62)	1301/2539	51.2 (41.3–61.2)	9 (1–65)	1371/2675	51.3 (41.6–60.9)	9 (1–65)
Decreased appetite	52/136	38.2 (27.3–50.2)	10 (3–39)	865/2540	34.1 (26.0–42.9)	11 (1–66)	917/2676	34.3 (26.3–42.9)	11 (1–66)
Feverishness	44/136	32.4 (20.2–46.5)	9 (1–38)	857/2538	33.8 (23.5–45.3)	7 (1–63)	901/2674	33.7 (23.5–45.2)	7 (1–63)
Fever (≥38.0°C)	26/136	19.1 (10.4–30.8)	6 (1–36)	524/2535	20.7 (14.7–27.8)	4 (1–63)	550/2671	20.6 (14.6–27.7)	4 (1–63)
Both upper respiratory symptoms and systemic symptoms/signs	110/136	80.9 (67.6–90.5)	…	2053/2541	80.8 (70.9–88.5)	…	2163/2677	80.8 (71.1–88.3)	…
Both lower respiratory symptoms/signs and systemic symptoms/signs	118/136	86.8 (74.3–94.6)	…	2180/2542	85.8 (77.5–91.9)	…	2298/2678	85.8 (77.6–91.9)	…
Other symptoms	14/136	10.3 (0.9–35.3)	…	421/2545	16.5 (4.0–39.3)	…	435/2681	16.2 (3.9–39.1)	…

Occurrence of symptoms/signs is shown as mean percentage and 95% exact CI accounting for clustered data. Duration is shown as median and minimum–maximum. Durations for “crackles (rales)/rhonchi,” “respiratory rate ≥20 breaths per minute,” “low or decreased oxygen saturation,” and “need for oxygen supplementation” are not provided, since these signs were assessed by the investigator at Visit 1.

Due to typographical data entry errors for 12 participants (2 cRSV-ARI, 10 non-cRSV-ARI), discovered after database lock, incorrect or unrealistic maximum durations are reported for “arthralgia,” “myalgia,” “nasal congestion,” and “sputum production.”

Abbreviations: ARI, acute respiratory infection; CI, confidence interval; cRSV-ARI, confirmed RSV-ARI; n, number of participants reporting symptom or sign; N, number of participants with complete start and end dates; RSV, respiratory syncytial virus; RT-PCR, reverse transcription polymerase chain reaction; …, no data.

^a^New or worsening.

The median duration of respiratory symptoms in the cRSV-ARI group ranged between 2 days (wheezing) and 19 days (sputum production and cough), while in the non-cRSV-ARI group, median durations were between 10 days (sore throat) to 16 days (sputum production and cough). Individual systemic symptoms had a median duration between 4 days (fever ≥38°C) and 17 days (fatigue) (Table 2). The overall highest median duration (≥14 days) was reported for nasal congestion/rhinorrhea, cough, sputum production, and fatigue.

### Prevalence of RSV-LRTD

**Figure 4.**
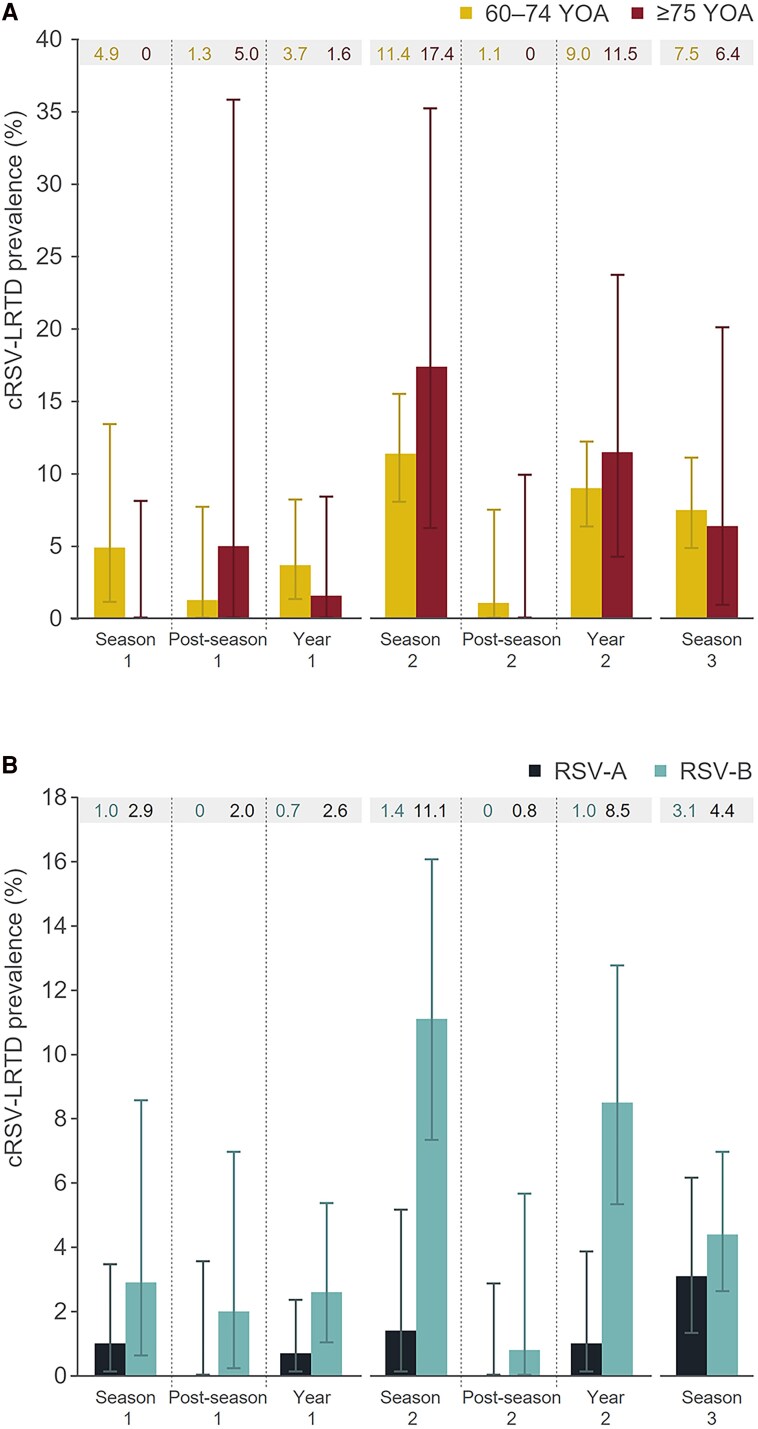
Prevalence estimates of RT-PCR confirmed RSV-LRTD (analysis set) by age category (*A*) and RSV subtype (*B*). Numbers on shaded background are the point estimates. Further details are shown in Supplementary Table 5 in the Supplementary material. Season 1, 01 October 2021 to 30 April 2022; Post-season 1, 01 May 2022 to 30 September 2022; Year 1, Season 1 and post-season 1; Season 2, 01 October 2022 to 30 April 2023; Post-season 2, 01 May 2023 to 30 September 2023; Year 2, Season 2 and Post-season 2; Season 3, 01 October 2023 to 30 April 2024. cRSV-LRTD, confirmed RSV-LRTD; LRTD, lower respiratory tract disease; RSV, respiratory syncytial virus; RT-PCR, reverse transcription polymerase chain reaction; YOA, years of age.

cRSV-LRTD prevalence by season and by strain showed similar trends as cRSV-ARI. The overall prevalence of cRSV-LRTD episodes during Year 1 was 3.3% (95% CI: 1.1–7.2), with most episodes (8/10) occurring during RSV Season 1 (3.9% [0.8–10.9]), and mostly (8/10) caused by RSV-B (2.6% [1.0–5.4]) (Figure 4; Supplementary Table 5). cRSV-LRTD prevalence increased during Year 2 to 9.5% (6.5–13.4), mostly due to increased prevalence during Season 2 (12.5% [9.1–16.7]), with most (41/46) infections also caused by RSV-B (8.5% [5.3–12.8]). Season 3 showed a decreased prevalence (7.3%; [4.9–10.3]), with slightly more RSV-LRTD infections (17/28) caused by RSV-B (4.4% [2.6–7.0]) vs RSV-A (3.1% [1.3–6.2]). Prevalence by country and setting is shown in Supplementary Table 6.

### Comorbidities

Predefined cardiorespiratory and endocrinometabolic comorbidities of interest were reported by 52.2% (71/136) of participants with cRSV-ARI. These included 20 (14.7%) participants reporting asthma, 14 (10.3%) participants reporting COPD, and 31 (22.8%) participants reporting type 2 diabetes mellitus. Among participants with non-cRSV-ARI, 39.3% (1006/2559) reported comorbidities of interest, including 298 (11.6%) participants reporting asthma, 252 (9.8%) participants reporting COPD, and 418 (16.3%) participants reporting type 2 diabetes mellitus. Predefined comorbidities of interest by age group are presented in Supplementary Table 7.

### Duration of ARI Episodes, Complications, and Coinfections

ARI episodes were resolved in 94.1% (128/136) of participants with cRSV-ARI, with a median duration of 21 days, vs 91.4% (2340/2559) of participants with non-cRSV-ARI with a median duration of 19 days (Table 3). More than half of the cRSV-ARI episodes (75/136, 55.1%) were considered LRTD by the investigator, and six (4.4%) led to complications without fatal outcomes. Of the non-cRSV-ARI episodes, less than half (1135/2559, 44.4%) were considered LRTD, and 81 (3.2%) led to complications with 4 (0.2%) fatal outcomes.

**Table 3.** Duration of ARI Episodes, Health Outcomes and Complications of the ARI Episodes, Combined for Year 1, Year 2 and Season 3 (Analysis Set)

	cRSV-ARI N = 136		Non-cRSV-ARI N = 2559		Overall N = 2695	
	Value or n	%	Value or n	%	Value or n	%
ARI outcome						
Recovered/Resolved	128	94.1	2340	91.4	2468	91.6
Recovering/Resolving	3	2.2	79	3.1	82	3.0
Not recovered/Not resolved	5	3.7	133	5.2	138	5.1
Recovered/Resolved with sequelae	0	0.0	3	0.1	3	0.1
Fatal	0	0.0	4	0.2	4	0.1
Median duration of ARI^a^, days (minimum–maximum)	21 (5–62)		19 (3–66)		19 (3–66)	
Is ARI considered LRTD?						
Yes	75	55.1	1135	44.4	1210	44.9
No	61	44.9	1424	55.6	1485	55.1
LRTD diagnosis						
Bronchitis	49	65.3	806	71.0	855	70.7
Pneumonia	4	5.3	45	4.0	49	4.0
Exacerbation of asthma	4	5.3	54	4.8	58	4.8
Exacerbation of COPD	10	13.3	115	10.1	125	10.3
Other LRTD	8	10.7	115	10.1	123	10.2
Any complication of ARI episode?						
Yes	6	4.4	81	3.2	87	3.2
No	130	95.6	2478	96.8	2608	96.8
Complication leading to hospitalization?						
Yes	1	0.7	24	0.9	25	0.9
No	135	99.3	2535	99.1	2670	99.1
Number of ER visits						
0	113	83.1	2193	85.7	2306	85.6
1	23	16.9	342	13.4	365	13.5
≥2	0	0.0	24	0.9	24	0.9
Participant admitted to ICU?						
Yes	0	0.0	3	0.1	3	0.1
No	136	100	2556	99.9	2692	99.9

Abbreviations: ARI, acute respiratory infection; COPD, chronic obstructive pulmonary disease; cRSV-ARI, confirmed RSV-ARI; ER, emergency room; ICU, intensive care unit; LRTD, lower respiratory tract disease; N, number of participants; n, number of participants in a given category; RSV, respiratory syncytial virus; RT-PCR, reverse transcription polymerase chain reaction.

^a^Including complications; only calculated for participants with complete onset and end dates.

Overall, 98 complications were reported (cRSV-ARI: 6; non-cRSV-ARI: 92), most of which were resolved (cRSV-ARI: 5/6 [83.3%]; non-cRSV-ARI: 73/92 [79.3%]). The most frequently reported complications were pneumonia (cRSV-ARI: 1/6 [16.7%]; non-cRSV-ARI 18/92 [19.6%]) and exacerbation of COPD (cRSV-ARI: 1/6 [16.7%]; non-cRSV-ARI: 17/92 [18.5%]). Hospitalization was required for 1/6 (16.7%) complications in the cRSV-ARI group and 29/92 (31.5%) complications in the non-cRSV-ARI group.

Infection with respiratory viruses other than RSV was detected in 61.3% (397/648) of participants during Year 1, 58.5% (623/1065) of participants during Year 2, and 52.0% (511/982) of participants during Season 3. Coinfection with RSV and other respiratory viruses in participants with cRSV-ARI occurred in 4/19 (21.1%) participants during Year 1, 14/76 (18.4%) participants during Year 2, and 7/41 (17.1%) participants during Season 3. These coinfections were mostly due to SARS-CoV-2 (Year 1: 2/19 [10.5%]; Year 2: 8/76 [10.5%]; Season 3: 4/41 [9.8%]; Supplementary Table 8).

## DISCUSSION

We found a seasonal prevalence estimate of cRSV-ARI of 3.6% during Season 1 (October 2021 to April 2022), which increased to 9.3% in Season 2, and decreased again to 4.2% in Season 3. Post-season prevalence was substantially lower (1.4% post-season 1% and 0.7% post-season 2). These findings are in line with previously reported proportions of RSV infection among adults ≥60 years of age with symptomatic respiratory infections, ranging from 0.0% to 21.5% in annual studies, with a pooled estimate of 4.7%. In seasonal studies, the pooled estimate increases to 7.8% [15]. However, the true prevalence is likely higher, due to limitations in testing methodology, the use of single-specimen vs paired specimens, and timing of specimen collection with regards to onset of symptoms [8]. We have tried to mitigate some of these limitations by using RT-PCR on nasopharyngeal swabs, and by increasing the testing window from seven to ten days. Since study participants were recruited among patients seeking medical assistance, our results do not provide an insight into the non-medically attended RSV prevalence or hospitalized RSV cases.

Following the COVID-19 pandemic, a shift in RSV seasonality was reported [16]. Due to the nonpharmaceutical interventions and social restrictions during the pandemic, and their gradual lifting toward the end of the pandemic, the peak of RSV infections during the 2021–2022 season occurred earlier than expected with a “rebound” effect during the following year [17–19]. Our observations show a similar effect with the highest prevalence observed during Season 2 (01 October 2022 to 30 April 2023).

Since the participants in our study were seeking medical advice for ARI, unsurprisingly, most participants reported upper respiratory symptoms as well as lower respiratory and systemic signs or symptoms. Clinically diagnosing RSV infection in adults, without laboratory confirmation is difficult, as shown by our observation that only sputum production and dyspnea showed a difference >10% between participants with cRSV-ARI and participants with non-cRSV-ARI. Furthermore, the median symptom duration can exceed 2 weeks, underscoring the substantial morbidity of RSV and emphasizing the need for ongoing surveillance and prevention. The proportion of LRTD in the cRSV-ARI group was higher compared to the non-cRSV-ARI group. Most of the complications were respiratory (pneumonia, exacerbation of COPD) and approximately 30% of the ARI cases with complications required hospitalization (cRSV-ARI: 1/6 [16.7%]; non-cRSV-ARI: 29/92 [31.5%]). Since many of the non-cRSV-ARI cases tested positive for other viral respiratory pathogens, such as SARS-CoV-2 and influenza, morbidity and mortality rates in the comparator group might be skewed upwards.

Over the three seasons of our study, co-infections with other viruses were mostly uniform. However, SARS-CoV-2 infections show a marked reduction over the course of our study (approximately 10% per season), while influenza and human rhinovirus infections showed increases during Year 2 and Season 3,compared to Season 1. This finding clearly shows the impact of the COVID-19 pandemic on the epidemiology of respiratory viruses and the shift in RSV seasonality over the following seasons [17–20]. The minor imbalance in unresolved and fatal ARI episodes could be the result of coinfections with influenza, or SARS-CoV-2, but the number of participants in these categories is too low to draw any conclusions from this observation.

A limitation of the current study is the limited recruitment during Year 1, combined with the changes in the study protocol before the start of Year 2. This low recruitment was likely caused by the disruptions of the health care systems during, and immediately after, the COVID-19 pandemic. The non-pharmaceutical interventions and restrictions during the pandemic also impacted the circulation of other respiratory viruses. In addition, the sample size did not allow us to reach any conclusion on RSV subtype dominance, as shown by the overlapping CIs in Seasons 1 and 3, or to perform robust analyses on the re-enrolled cases. The high proportion of other viral infections that also impact morbidity and mortality in the comparator non-cRSV-ARI group makes it challenging to evaluate the true impact of RSV. Finally, to reduce the burden on the study participants and study sites, we only used nasopharyngeal swabs. As previously reported, RSV detection increases by a factor of 1.4–1.6 when using paired serology and sputum [21] and additional samples [8, 22]. It is therefore likely we underestimated the true RSV prevalence in our study.

The strengths of our study are its duration, the sample size, and geographical distribution over six European countries. By running the study over three consecutive RSV seasons, including the interseasonal periods for continuous recruitment, we believe our results are representative. Furthermore, the addition of a third season allowed us to further investigate the shifted, postpandemic epidemiology of RSV and show that RSV seasonality in Europe has returned to pre-pandemic trends, with substantial country-level variations.

Our findings confirm the substantial burden of RSV in older adults and highlight the need for further awareness among patients and health care professionals, as well as the need for national surveillance programs at the RSV subtype level.

## Supplementary Material

ciag146_Supplementary_Data

## Supplementary Data

Supplementary materials are available at *Clinical Infectious Diseases* online. Consisting of data provided by the authors to benefit the reader, the posted materials are not copyedited and are the sole responsibility of the authors, so questions or comments should be addressed to the corresponding author.
