# Supplementary material for: Burden of Respiratory Syncytial Virus Among Older Adults With an Acute Respiratory Infection: A Prospective Study in Six European Countries
Source: Clin Infect Dis. 2026 Mar 6;82(6):e1266–76. doi: 10.1093/cid/ciag146 (PMC13341239; doi:10.1093/cid/ciag146)
Supplement: ciag146_Supplementary_Data [file ciag146_supplementary_data.docx]

Burden of respiratory syncytial virus among older adults with an acute respiratory infection: a prospective study in six European countries.

# Supplemental material

## Table of contents

[Case definitions 2](#_Toc210220010)

[Table S1. Case definition of acute respiratory infection symptoms or signs 2](#_Toc210220011)

[Inclusion / Exclusion criteria 3](#_Toc210220012)

[Table S2. Study objectives and endpoints. 4](#_Toc210220013)

[Table S3. Prevalence estimates of RT-PCR confirmed RSV-ARI (analysis sets) overall, by age group and RSV subtype 5](#_Toc210220014)

[Table S4. Prevalence estimates of cRSV-ARI (analysis sets) by country and setting 6](#_Toc210220015)

[Table S5. Prevalence estimates of RT-PCR confirmed RSV-LRTD (analysis sets) 8](#_Toc210220016)

[Table S6. Prevalence estimates of cRSV-LRTD (analysis sets) by country and setting 9](#_Toc210220017)

[Table S7. Predefined comorbidities of interest by age group (analysis set) 11](#_Toc210220018)

[Table S8. Prevalence of respiratory viruses other than RSV 12](#_Toc210220019)

[References 15](#_Toc210220020)

Case definitions

An episode of acute respiratory infection is defined as:

- Presence of at least 2 respiratory symptoms or signs for at least 24 hours

OR

- Presence of at least 1 respiratory symptom or sign AND 1 systemic symptom or sign for at least 24 hours.

An episode of lower respiratory tract disease is defined as:

- Presence of at least 2 lower respiratory symptoms or signs for at least 24 hours, including at least 1 lower respiratory sign

OR

- Presence of at least 3 lower respiratory symptoms for at least 24 hours.

Table S1. Case definition of acute respiratory infection symptoms or signs

| **ARI Symptom or sign** | **Description** |
| --- | --- |
| Upper respiratory symptoms | - Nasal congestion/rhinorrhea - Sore throat |
| Lower respiratory symptoms | - New or increased sputum - New or increased cough - New or increased dyspnea (shortness of breath) |
| Lower respiratory signs | - New or increased wheezing ^a^ - New or increased crackles (rales) / rhonchi, based on chest auscultation ^b^ - Respiratory rate ≥20 respirations/minute ^b^ - Low or decreased oxygen saturation (O_2_ saturation <95%, or ≤90% if baseline is <95%) ^b^ - Need for oxygen supplementation ^a^ |
| Systemic signs and symptoms | - Fever / Feverishness ^c^ - Fatigue - Body aches (myalgia / arthralgia) - Headache - Decreased appetite |

^a^ Reported by study participant or investigator

^b^ Measured and reported by investigator only at visit 1

^c^ Fever is defined as a temperature ≥38°C / 100.4°F by any route; Feverishness is defined as the feeling of having a fever, without objective measurement.

ARI, acute respiratory infection.

Inclusion / Exclusion criteria

All participants must satisfy **ALL** of the following criteria at study entry:

- A male or female aged ≥60 years at onset of the acute respiratory infection (ARI).
- Participants who were diagnosed, at the time of enrollment, with at least 2 ARI symptoms or signs meeting the ARI case definition for at least 24 hours and an onset date of first symptom or sign less than 10* days before the medical visit at the general practitioner or outpatient clinic or hospital.
  ** Note: This time window was extended from 7 days to 10 days following protocol amendment 2.*
- Participants who, in the opinion of the investigator, could and would comply with the requirements of the study procedures (e.g., agree to provide nasal and throat swab samples, complete self-administered questionnaires, attend regular phone calls, etc.).
  *Note: In case of physical incapacity that would preclude the self-completion of the diary cards and/or questionnaires, either site staff could assist the participant (for activities performed during site visits) or the participant could assign a caregiver to assist him/her with this activity (for activities performed at home). However, at no time, the site staff or caregiver were to evaluate the participant’s health status while answering diaries and/or questionnaires or make decisions on behalf of the participant.*
- Participants who were able to provide written informed consent.

The following criteria were checked at the time of study entry. If **ANY** of the exclusion criteria applied, the participant **was not** included in the study.

- History of vaccination with an investigational or licensed respiratory syncytial virus (RSV) vaccine and planned administration of any RSV drugs or RSV vaccine at any time during the study period.
  *Note: Any RSV drug includes any investigational, preventive or curative drug targeting RSV specifically (including biological products such as vaccines).*
- Participants with a known cognitive impairment (based on the medical judgment of the investigator).
- Re-enrollment if new ARI symptoms or signs were reported with 7 days from the resolution of the previous ARI or before end of follow-up of the previous ARI (Day 29 if ARI resolved or Day 57 if ARI not resolved by Day 29).

Table S2. Study objectives and endpoints

| **Objectives** | **Endpoints** |
| --- | --- |
| **Primary** | |
| To estimate the prevalence of RT-PCR confirmed RSV-ARI in participants, by season, by country, by age group, by setting and by RSV subtype (A/B). | Occurrence of RT-PCR confirmed RSV-ARI in combined nasal and throat swab samples of participants presenting with ARI, from samples collected at visit 1 (Day 1). |
| **Secondary** | |
| To describe duration and symptoms or signs of ARI in participants with RT-PCR confirmed RSV infection by season, by country, by age group, by setting, by RSV subtype (A/B) and overall. | Duration of ARI and occurrence of symptoms or signs of ARI in participants with RT-PCR confirmed RSV infection, during the 28 days after visit 1 (until Day 29) if ARI resolves within 4 weeks or for 56 days after visit 1 (until Day 57) if ARI has not resolved after 4 weeks. |
| To describe underlying comorbidities of participants with RT-PCR confirmed RSV infection. | Presence of comorbidities (past, current and chronic) in participants with RT-PCR confirmed RSV infection at visit 1 (Day 1). |
| To estimate the prevalence of RT-PCR confirmed RSV-LRTD in participants by season, by country, by age group, by setting and by RSV subtype (A/B). | Occurrence of RT-PCR confirmed RSV-LRTD in combined nasal and throat swab samples of participants with LRTD, from samples collected at visit 1 (Day 1). |
| To describe complications, hospitalizations and number of deaths associated with RT-PCR confirmed RSV infection by season, by country, by age group, by setting, by RSV subtype (A/B) and overall. | Occurrence of complications, hospitalizations and deaths for participants with RT-PCR confirmed RSV-ARI, during the 28 days after visit 1 (until Day 29) if ARI resolves within 4 weeks or for 56 days after visit 1 (until Day 57) if ARI has not resolved after 4 weeks. |
| To estimate the prevalence of co-infection with other respiratory viruses in participants presenting with RT-PCR confirmed RSV infection by season, by country, by age group, by setting and by RSV subtype (A/B). | Occurrence of other viral pathogens in combined nasal and throat swab samples of participants with RT-PCR confirmed RSV-ARI, from samples collected at visit 1 (Day 1). |

Note: additional secondary objectives related to the impact on health-related quality of life for participants, healthcare resource utilization, and days of work lost by participants and caregivers are reported in the accompanying manuscript [1].

ARI, acute respiratory infection; LRTD, lower respiratory tract disease; RSV, respiratory syncytial virus; RT-PCR, reverse transcription polymerase chain reaction.

Table S3. Prevalence estimates of RT-PCR confirmed RSV-ARI (analysis sets) overall, by age group and RSV subtype

|  | **n/N** | | **Prevalence** | **95% CI** | |  | **n/N** | | **Prevalence** | **95% CI** | |  | **n/N** | | **Prevalence** | **95% CI** | |
| --- | --- | --- | --- | --- | --- | --- | --- | --- | --- | --- | --- | --- | --- | --- | --- | --- | --- |
|  | **Season 1** | | | | |  | **Post-season 1** | | | | |  | **Year 1** | | | | |
| Overall | 16/440 | 3.6% | | | 1.4–7.5 |  | 3/208 | 1.4% | | | 0.3–4.5 |  | 19/648 | 2.9% | | | 1.5–5.0 |
| Age group ^a^ |  |  | | |  |  |  |  | | |  |  |  |  | | |  |
| 60–74 | 14/359 | 3.9% | | | 1.4–8.4 |  | 1/167 | 0.6% | | | 0.0–3.8 |  | 15/526 | 2.9% | | | 1.4–5.2 |
| ≥75 | 2/81 | 2.5% | | | 0.1–10.7 |  | 2/41 | 4.9% | | | 0.3–20.6 |  | 4/122 | 3.3% | | | 0.9–8.2 |
| RSV subtype |  |  | | |  |  |  |  | | |  |  |  |  | | |  |
| RSV-A | 4/440 | 0.9% | | | 0.2–2.3 |  | 1/208 | 0.5% | | | 0.0–3.5 |  | 5/648 | 0.8% | | | 0.3–1.8 |
| RSV-B | 12/440 | 2.7% | | | 1.0–5.8 |  | 2/208 | 1.0% | | | 0.1–3.4 |  | 14/648 | 2.2% | | | 1.2–3.7 |
|  | **Season 2** | | | | |  | **Post-season 2** | | | | |  | **Year 2** | | | | |
| Overall | 74/793 | 9.3% | | | 7.2–11.8 |  | 2/272 | 0.7% | | | 0.1–3.1 |  | 76/1065 | 7.1% | | | 5.3–9.4 |
| Age group |  |  | | |  |  |  |  | | |  |  |  |  | | |  |
| 60–74 | 53/628 | 8.4% | | | 6.4–10.9 |  | 2/198 | 1.0% | | | 0.1–4.2 |  | 55/826 | 6.7% | | | 5.0–8.7 |
| ≥75 | 21/165 | 12.7% | | | 6.0–22.8 |  | 0/74 | 0.0% | | | 0.0–4.9 |  | 21/239 | 8.8% | | | 4.0–16.2 |
| RSV subtype |  |  | | |  |  |  |  | | |  |  |  |  | | |  |
| RSV-A | 14/793 | 1.8% | | | 0.6–3.9 |  | 0/272 | 0.0% | | | 0.0–1.3 |  | 14/1065 | 1.3% | | | 0.4–3.0 |
| RSV-B | 60/793 | 7.6% | | | 5.3–10.5 |  | 2/272 | 0.7% | | | 0.1–3.1 |  | 62/1065 | 5.8% | | | 3.9–8.3 |
|  | **Season 3** | | | | |  |  |  | | |  |  |  |  | | |  |
| Overall | 41/982 | 4.2% | | | 2.7–6.1 |  |  |  | | |  |  |  |  | | |  |
| Age group |  |  | | |  |  |  |  | | |  |  |  |  | | |  |
| 60–74 | 32/728 | 4.4% | | | 3.0–6.1 |  |  |  | | |  |  |  |  | | |  |
| ≥75 | 9/254 | 3.5% | | | 0.6–10.6 |  |  |  | | |  |  |  |  | | |  |
| RSV Subtype |  |  | | |  |  |  |  | | |  |  |  |  | | |  |
| RSV-A | 21/982 | 2.1% | | | 0.9–4.3 |  |  |  | | |  |  |  |  | | |  |
| RSV-B | 21/982 | 2.1% | | | 1.3–3.3 |  |  |  | | |  |  |  |  | | |  |

^a^ Expressed in years, at ARI onset.

Season 1, 01 October 2021 to 30 April 2022; Post-season 1, 01 May 2022 to 30 September 2022; Year 1, Season 1 and post-season 1; Season 2, 01 October 2022 to 30 April 2023; Post-season 2, 01 May 2023 to 30 September 2023; Year 2, Season 2 and post-season 2; Season 3, 01 October 2023 to 30 April 2024.

ARI, acute respiratory infection; CI, confidence interval; N, number of participants; n, number of participants with RT-PCR confirmed RSV-ARI; RSV, respiratory syncytial virus; RT-PCR, reverse transcription polymerase chain reaction.

Table S4. Prevalence estimates of cRSV-ARI (analysis sets) by country and setting

|  | **n/N** | | **Prevalence** | **95% CI** | |  | **n/N** | | **Prevalence** | **95% CI** | |  | **n/N** | | **Prevalence** | **95% CI** | |
| --- | --- | --- | --- | --- | --- | --- | --- | --- | --- | --- | --- | --- | --- | --- | --- | --- | --- |
|  | **Season 1** | | | | |  | **Post-season 1** | | | | |  | **Year 1** | | | | |
| Country ^a^ |  |  | | |  |  |  |  | | |  |  |  |  | | |  |
| Finland | 3/29 | 10.3% | | | 1.7–29.9 |  | - | - | | | - |  | 3/29 | 10.3% | | | 1.7–29.9 |
| Germany | - | - | | | - |  | - | - | | | - |  | - | - | | | - |
| Italy | 1/77 | 1.3% | | | 0.0–8.6 |  | 1/16 | 6.3% | | | 0.0–100 |  | 2/93 | 2.2% | | | 0.0–87.7 |
| Poland | - | - | | | - |  | - | - | | | - |  | - | - | | | - |
| Spain | 8/261 | 3.1% | | | 0.5–9.9 |  | 2/122 | 1.6% | | | 0.2–6.0 |  | 10/383 | 2.6% | | | 1.1–5.1 |
| UK | 4/73 | 5.5% | | | 0.0–34.0 |  | 0/70 | 0.0% | | | 0.0–5.1 |  | 4/143 | 2.8% | | | 0.0–17.0 |
| Setting |  |  | | |  |  |  |  | | |  |  |  |  | | |  |
| GP | 8/315 | 2.5% | | | 1.1–4.9 |  | 2/129 | 1.6% | | | 0.2–5.5 |  | 10/444 | 2.3% | | | 1.1–4.1 |
| Outpatient clinic/hospital | 8/125 | 6.4% | | | 0.5–24.1 |  | 1/79 | 1.3% | | | 0.0–6.9 |  | 9/204 | 4.4% | | | 1.2–10.9 |
|  | **Season 2** | | | | |  | **Post-season 2** | | | | |  | **Year 2** | | | | |
| Country ^a^ |  |  | | |  |  |  |  | | |  |  |  |  | | |  |
| Finland | - | - | | | - |  | - | - | | | - |  | - | - | | | - |
| Germany | 10/54 | 18.5% | | | 9.3–31.4 |  | 0/3 | 0.0% | | | 0.0–70.8 |  | 10/57 | 17.5% | | | 8.7–29.9 |
| Italy | 13/112 | 11.6% | | | 6.3–19.2 |  | 0/15 | 0.0% | | | 0.0–21.8 |  | 13/127 | 10.2% | | | 5.6–16.9 |
| Poland | 24/270 | 8.9% | | | 5.4–13.6 |  | 1/87 | 1.1% | | | 0.0–9.3 |  | 25/357 | 7.0% | | | 3.6–12.1 |
| Spain | 19/235 | 8.1% | | | 4.9–12.3 |  | 1/136 | 0.7% | | | 0.0–6.8 |  | 20/371 | 5.4% | | | 2.9–9.0 |
| UK | 8/122 | 6.6% | | | 2.9–12.5 |  | 0/31 | 0.0% | | | 0.0–11.2 |  | 8/153 | 5.2% | | | 2.1–10.5 |
| Setting |  |  | | |  |  |  |  | | |  |  |  |  | | |  |
| GP | 51/498 | 10.2% | | | 7.1–14.2 |  | 2/162 | 1.2% | | | 0.0–7.2 |  | 53/660 | 8.0% | | | 5.2–11.8 |
| Outpatient clinic/hospital | 23/295 | 7.8% | | | 5.0–11.5 |  | 0/110 | 0.0% | | | 0.0–3.3 |  | 23/405 | 5.7% | | | 3.6–8.4 |

(table continues on next page)

|  | **n/N** | | **Prevalence** | **95% CI** | |  | **n/N** | | **Prevalence** | **95% CI** | |  | **n/N** | | **Prevalence** | **95% CI** | |
| --- | --- | --- | --- | --- | --- | --- | --- | --- | --- | --- | --- | --- | --- | --- | --- | --- | --- |
|  | **Season 3** | | | | |  |  |  | | |  |  |  |  | | |  |
| Country ^a^ |  |  | | |  |  |  |  | | |  |  |  |  | | |  |
| Finland | - | - | | | - |  |  |  | | |  |  |  |  | | |  |
| Germany | 1/77 | 1.3% | | | 0.0–7.0 |  |  |  | | |  |  |  |  | | |  |
| Italy | 2/130 | 1.5% | | | 0.0–57.1 |  |  |  | | |  |  |  |  | | |  |
| Poland | 14/377 | 3.7% | | | 1.2–8.5 |  |  |  | | |  |  |  |  | | |  |
| Spain | 19/329 | 5.8% | | | 3.1–9.8 |  |  |  | | |  |  |  |  | | |  |
| UK | 5/69 | 7.2% | | | 0.1–38.7 |  |  |  | | |  |  |  |  | | |  |
| Setting |  |  | | |  |  |  |  | | |  |  |  |  | | |  |
| GP | 31/660 | 4.7% | | | 3.2–6.6 |  |  |  | | |  |  |  |  | | |  |
| Outpatient clinic/hospital | 10/322 | 3.1% | | | 0.9–7.4 |  |  |  | | |  |  |  |  | | |  |

^a^ In Finland, recruitment ended at the end of Season 1 (30 April 2022). In Germany and Poland, recruitment started at the beginning of Season 2 (01 October 2022)

Season 1, 01 October 2021 to 30 April 2022; Post-season 1, 01 May 2022 to 30 September 2022; Year 1, Season 1 and post-season 1;
Season 2, 01 October 2022 to 30 April 2023; Post-season 2, 01 May 2023 to 30 September 2023; Year 2, Season 2 and post-season 2;
Season 3, 01 October 2023 to 30 April 2024.

CI, exact confidence interval accounting for clustered data; cRSV-ARI, RT-PCR confirmed RSV acute respiratory infection; GP, general practitioner; N, number of participants; n, number of participants with cRSV-ARI; RSV, respiratory syncytial virus; RT-PCR, reverse transcription polymerase chain reaction; UK, United Kingdom.

Table S5. Prevalence estimates of RT-PCR confirmed RSV-LRTD (analysis sets)

|  | **n/N** | | **Prevalence** | **95% CI** | |  | **n/N** | | **Prevalence** | **95% CI** | |  | **n/N** | | **Prevalence** | **95% CI** | |
| --- | --- | --- | --- | --- | --- | --- | --- | --- | --- | --- | --- | --- | --- | --- | --- | --- | --- |
|  | **Season 1** | | | | |  | **Post-season 1** | | | | |  | **Year 1** | | | | |
| Overall | 8/206 | 3.9% | | | 0.8–10.9 |  | 2/100 | 2.0% | | | 0.2–7.0 |  | 10/306 | 3.3% | | | 1.1–7.2 |
| Age group ^a^ |  |  | | |  |  |  |  | | |  |  |  |  | | |  |
| 60–74 | 8/163 | 4.9% | | | 1.1–13.5 |  | 1/80 | 1.3% | | | 0.0–7.8 |  | 9/243 | 3.7% | | | 1.3–8.3 |
| ≥75 | 0/43 | 0.0% | | | 0.0–8.2 |  | 1/20 | 5.0% | | | 0.0–35.9 |  | 1/63 | 1.6% | | | 0.0–8.5 |
| RSV subtype |  |  | | |  |  |  |  | | |  |  |  |  | | |  |
| RSV-A | 2/206 | 1.0% | | | 0.1–3.5 |  | 0/100 | 0.0% | | | 0.0–3.6 |  | 2/306 | 0.7% | | | 0.1–2.4 |
| RSV-B | 6/206 | 2.9% | | | 0.6–8.6 |  | 2/100 | 2.0% | | | 0.2–7.0 |  | 8/306 | 2.6% | | | 1.0–5.4 |
|  | **Season 2** | | | | |  | **Post-season 2** | | | | |  | **Year 2** | | | | |
| Overall | 45/359 | 12.5% | | | 9.1–16.7 |  | 1/124 | 0.8% | | | 0.0–5.7 |  | 46/483 | 9.5% | | | 6.5–13.4 |
| Age group |  |  | | |  |  |  |  | | |  |  |  |  | | |  |
| 60–74 | 33/290 | 11.4% | | | 8.0–15.6 |  | 1/89 | 1.1% | | | 0.0–7.6 |  | 34/379 | 9.0% | | | 6.3–12.3 |
| ≥75 | 12/69 | 17.4% | | | 6.2–35.3 |  | 0/35 | 0.0% | | | 0.0–10.0 |  | 12/104 | 11.5% | | | 4.2–23.8 |
| RSV subtype |  |  | | |  |  |  |  | | |  |  |  |  | | |  |
| RSV-A | 5/359 | 1.4% | | | 0.1–5.2 |  | 0/124 | 0.0% | | | 0.0–2.9 |  | 5/483 | 1.0% | | | 0.1–3.9 |
| RSV-B | 40/359 | 11.1% | | | 7.3–16.1 |  | 1/124 | 0.8% | | | 0.0–5.7 |  | 41/483 | 8.5% | | | 5.3–12.8 |
|  | **Season 3** | | | | |  |  |  | | |  |  |  |  | | |  |
| Overall | 28/386 | 7.3% | | | 4.9–10.3 |  |  |  | | |  |  |  |  | | |  |
| Age group |  |  | | |  |  |  |  | | |  |  |  |  | | |  |
| 60–74 | 22/292 | 7.5% | | | 4.8–11.2 |  |  |  | | |  |  |  |  | | |  |
| ≥75 | 6/94 | 6.4% | | | 0.9–20.2 |  |  |  | | |  |  |  |  | | |  |
| RSV Subtype |  |  | | |  |  |  |  | | |  |  |  |  | | |  |
| RSV-A | 12/386 | 3.1% | | | 1.3–6.2 |  |  |  | | |  |  |  |  | | |  |
| RSV-B | 17/386 | 4.4% | | | 2.6–7.0 |  |  |  | | |  |  |  |  | | |  |

^a^ Expressed in years, at ARI onset.

Season 1, 01 October 2021 to 30 April 2022; Post-season 1, 01 May 2022 to 30 September 2022; Year 1, Season 1 and post-season 1; Season 2, 01 October 2022 to 30 April 2023; Post-season 2, 01 May 2023 to 30 September 2023; Year 2, Season 2 and post-season 2; Season 3, 01 October 2023 to 30 April 2024.

ARI, acute respiratory infection; CI, confidence interval; LRTD, lower respiratory tract disease; N, number of participants; n, number of participants with RT-PCR confirmed RSV-LRTD; RSV, respiratory syncytial virus; RT-PCR, reverse transcription polymerase chain reaction.

Table S6. Prevalence estimates of cRSV-LRTD (analysis sets) by country and setting

|  | **n/N** | | **Prevalence** | **95% CI** | |  | **n/N** | | **Prevalence** | **95% CI** | |  | **n/N** | | **Prevalence** | **95% CI** | |
| --- | --- | --- | --- | --- | --- | --- | --- | --- | --- | --- | --- | --- | --- | --- | --- | --- | --- |
|  | **Season 1** | | | | |  | **Post-season 1** | | | | |  | **Year 1** | | | | |
| Country ^a^ |  |  | | |  |  |  |  | | |  |  |  |  | | |  |
| Finland | 1/8 | 12.5% | | | 0.0–95.8 |  | - | - | | | - |  | 1/8 | 12.5% | | | 0.0–95.8 |
| Germany | - | - | | | - |  | - | - | | | - |  | - | - | | | - |
| Italy | 0/22 | 0.0% | | | 0.0–15.4 |  | 0/1 | 0.0% | | | 0.0–97.5 |  | 0/23 | 0.0% | | | 0.0–14.8 |
| Poland | - | - | | | - |  | - | - | | | - |  | - | - | | | - |
| Spain | 4/136 | 2.9% | | | 0.2–11.9 |  | 2/62 | 3.2% | | | 0.4–11.5 |  | 6/198 | 3.0% | | | 1.0–7.0 |
| UK | 3/40 | 7.5% | | | 0.0–52.3 |  | 0/37 | 0.0% | | | 0.0–9.5 |  | 3/77 | 3.9% | | | 0.0–27.4 |
| Setting |  |  | | |  |  |  |  | | |  |  |  |  | | |  |
| GP | 3/132 | 2.3% | | | 0.0–14.7 |  | 2/61 | 3.3% | | | 0.4–11.3 |  | 5/193 | 2.6% | | | 0.3–8.7 |
| Outpatient clinic/hospital | 5/74 | 6.8% | | | 0.2–31.2 |  | 0/39 | 0.0% | | | 0.0–9.0 |  | 5/113 | 4.4% | | | 0.3–17.4 |
|  | **Season 2** | | | | |  | **Post-season 2** | | | | |  | **Year 2** | | | | |
| Country ^a^ |  |  | | |  |  |  |  | | |  |  |  |  | | |  |
| Finland | - | - | | | - |  | - | - | | | - |  | - | - | | | - |
| Germany | 6/30 | 20.0% | | | 7.7–38.6 |  | - | - | | | - |  | 6/30 | 20.0% | | | 7.7–38.6 |
| Italy | 5/20 | 25.0% | | | 5.0–58.7 |  | 0/1 | 0.0% | | | 0.0–97.5 |  | 5/21 | 23.8% | | | 4.5–57.2 |
| Poland | 17/100 | 17.0% | | | 6.4–33.5 |  | 0/24 | 0.0% | | | 0.0–14.2 |  | 17/124 | 13.7% | | | 4.3–30.0 |
| Spain | 12/130 | 9.2% | | | 4.9–15.6 |  | 1/76 | 1.3% | | | 0.0–12.0 |  | 13/206 | 6.3% | | | 2.8–11.9 |
| UK | 5/79 | 6.3% | | | 2.1–14.2 |  | 0/23 | 0.0% | | | 0.0–14.8 |  | 5/102 | 4.9% | | | 1.6–11.1 |
| Setting |  |  | | |  |  |  |  | | |  |  |  |  | | |  |
| GP | 35/248 | 14.1% | | | 9.2–20.3 |  | 1/77 | 1.3% | | | 0.0–12.9 |  | 36/325 | 11.1% | | | 6.3–17.7 |
| Outpatient clinic/hospital | 10/111 | 9.0% | | | 4.0–16.8 |  | 0/47 | 0.0% | | | 0.0–7.5 |  | 10/158 | 6.3% | | | 3.0–11.5 |

(table continues on next page)

|  | **n/N** | | **Prevalence** | **95% CI** | |  | **n/N** | | **Prevalence** | **95% CI** | |  | **n/N** | | **Prevalence** | **95% CI** | |
| --- | --- | --- | --- | --- | --- | --- | --- | --- | --- | --- | --- | --- | --- | --- | --- | --- | --- |
|  | **Season 3** | | | | |  |  |  | | |  |  |  |  | | |  |
| Country ^a^ |  |  | | |  |  |  |  | | |  |  |  |  | | |  |
| Finland | - | - | | | - |  |  |  | | |  |  |  |  | | |  |
| Germany | 1/43 | 2.3% | | | 0.1–12.3 |  |  |  | | |  |  |  |  | | |  |
| Italy | 0/12 | 0.0% | | | 0.0–26.5 |  |  |  | | |  |  |  |  | | |  |
| Poland | 6/105 | 5.7% | | | 2.1–12.0 |  |  |  | | |  |  |  |  | | |  |
| Spain | 16/183 | 8.7% | | | 4.6–14.8 |  |  |  | | |  |  |  |  | | |  |
| UK | 5/43 | 11.6% | | | 1.5–36.0 |  |  |  | | |  |  |  |  | | |  |
| Setting |  |  | | |  |  |  |  | | |  |  |  |  | | |  |
| GP | 21/293 | 7.2% | | | 4.5–10.7 |  |  |  | | |  |  |  |  | | |  |
| Outpatient clinic/hospital | 7/93 | 7.5% | | | 1.0–24.1 |  |  |  | | |  |  |  |  | | |  |

^a^ In Finland, recruitment ended at the end of Season 1 (30 April 2022). In Germany and Poland, recruitment started at the beginning of Season 2 (01 October 2022)

Season 1, 01 October 2021 to 30 April 2022; Post-season 1, 01 May 2022 to 30 September 2022; Year 1, Season 1 and post-season 1;
Season 2, 01 October 2022 to 30 April 2023; Post-season 2, 01 May 2023 to 30 September 2023; Year 2, Season 2 and post-season 2;
Season 3, 01 October 2023 to 30 April 2024.

CI, exact confidence interval accounting for clustered data; cRSV-LRTD, RT-PCR confirmed RSV lower respiratory tract disease; GP, general practitioner; N, number of participants; n, number of participants with cRSV-LRTD; RSV, respiratory syncytial virus; RT-PCR, reverse transcription polymerase chain reaction; UK, United Kingdom.

Table S7. Predefined comorbidities of interest by age group (analysis set)

|  | **cRSV-ARI** | | **Non-cRSV-ARI** | | **Overall** | |
| --- | --- | --- | --- | --- | --- | --- |
|  | **n/N** | **%** | **n/N** | **%** | **n/N** | **%** |
| **60–74 years of age** |  |  |  |  |  |  |
| Cardiorespiratory conditions |  |  |  |  |  |  |
| COPD | 6/102 | 5.9% | 207/1978 | 10.5% | 213/2080 | 10.2% |
| Asthma | 16/102 | 15.7% | 239/1978 | 12.1% | 255/2080 | 12.3% |
| Any chronic respiratory/pulmonary disease ^a^ | 23/102 | 22.5% | 441/1978 | 22.3% | 464/2080 | 22.3% |
| Chronic heart failure | 3/102 | 2.9% | 31/1978 | 1.6% | 34/2080 | 1.6% |
| Endocrinometabolic conditions |  |  |  |  |  |  |
| Diabetes mellitus type 1 or type 2 | 21/102 | 20.6% | 294/1978 | 14.9% | 315/2080 | 15.1% |
| Type 2 diabetes mellitus^b^ | 21/102 | 20.6% | 286/1978 | 14.5% | 307/2080 | 14.8% |
| Type 1 diabetes mellitus^b^ | 0/102 | 0.0% | 7/1978 | 0.4% | 7/2080 | 0.3% |
| Advanced liver or renal disease | 7/102 | 6.9% | 80/1978 | 4.0% | 87/2080 | 4.2% |
| **≥75 years of age** |  |  |  |  |  |  |
| Cardiorespiratory conditions |  |  |  |  |  |  |
| COPD | 9/34 | 26.5% | 96/581 | 16.5% | 105/615 | 17.1% |
| Asthma | 4/34 | 11.8% | 72/581 | 12.4% | 76/615 | 12.4% |
| Any chronic respiratory/pulmonary disease ^a^ | 13/34 | 38.2% | 171/581 | 29.4% | 184/615 | 29.9% |
| Chronic heart failure | 6/34 | 17.6% | 36/581 | 6.2% | 42/615 | 6.8% |
| Endocrinometabolic conditions |  |  |  |  |  |  |
| Diabetes mellitus type 1 or type 2 | 10/34 | 29.4% | 138/581 | 23.8% | 148/615 | 24.1% |
| Type 2 diabetes mellitus ^b^ | 10/34 | 29.4% | 132/581 | 22.7% | 142/615 | 23.1% |
| Type 1 diabetes mellitus ^b^ | NA | NA | NA | NA | NA | NA |
| Advanced liver or renal disease | 2/34 | 5.9% | 44/581 | 7.6% | 46/615 | 7.5% |

Note: the comorbidities of interest are not mutually exclusive.

^a^ This comorbidity of interest also includes asthma and COPD.

^b^ Type 1 and Type 2 diabetes mellitus were not individually predefined as separate comorbidities of interest. The values shown are at the Preferred Term level.

COPD; chronic obstructive pulmonary disease; cRSV-ARI, RT-PCR confirmed RSV acute respiratory infection; N, number of participants; n/%, number/percentage of participants in the specified category; NA, not available; RSV, respiratory syncytial virus; RT-PCR, reverse transcription polymerase chain reaction.

Table S8. Prevalence of respiratory viruses other than RSV

|  | **cRSV-ARI N=136** | | **Non-cRSV-ARI N=2559** | | **Overall N=2695** | |
| --- | --- | --- | --- | --- | --- | --- |
|  | **n/N** | **% (95% CI)** | **n/N** | **% (95% CI)** | **n/N** | **% (95% CI)** |
| **Year 1** |  |  |  |  |  |  |
| Any respiratory viruses | 4/19 | 21.1 (5.3–47.9) | 393/629 | 62.5 (43.2–79.2) | 397/648 | 61.3 (42.9–77.6) |
| Any influenza A or B virus | 1/19 | 5.3 (0.0–33.4) | 39/629 | 6.2 (3.8–9.5) | 40/648 | 6.2 (3.8–9.5) |
| Any influenza A subtype | 1/19 | 5.3 (0.0–33.4) | 39/629 | 6.2 (3.8–9.5) | 40/648 | 6.2 (3.8–9.5) |
| Human influenza A virus | 0/19 | 0.0 (0.0–17.6) | 30/629 | 4.8 (2.8–7.5) | 30/648 | 4.6 (2.7–7.3) |
| subtype H1pdm09 | 1/19 | 5.3 (0.0–33.4) | 2/629 | 0.3 (0.0–1.2) | 3/648 | 0.5 (0.1–1.7) |
| subtype H3 | 0/19 | 0.0 (0.0–17.6) | 36/629 | 5.7 (3.2–9.4) | 36/648 | 5.6 (3.1–9.2) |
| Human adenovirus | 0/19 | 0.0 (0.0–17.6) | 2/629 | 0.3 (0.0–1.3) | 2/648 | 0.3 (0.0–1.2) |
| Human metapneumovirus | 0/19 | 0.0 (0.0–17.6) | 12/629 | 1.9 (1.0-3.3) | 12/648 | 1.9 (1.0–3.2) |
| Human enterovirus | 0/19 | 0.0 (0.0–17.6) | 4/629 | 0.6 (0.2–1.6) | 4/648 | 0.6 (0.2–1.6) |
| Any human parainfluenzavirus | 2/19 | 10.5 (1.3–33.1) | 23/629 | 3.7 (1.4–7.7) | 25/648 | 3.9 (1.6–7.8) |
| Human parainfluenzavirus 1 | - | - | - | - | - | - |
| Human parainfluenzavirus 2 | 0/19 | 0.0 (0.0–17.6) | 2/629 | 0.3 (0.0–1.3) | 2/648 | 0.3 (0.0–1.2) |
| Human parainfluenzavirus 3 | 1/19 | 5.3 (0.1–26.0) | 16/629 | 2.5 (0.8–5.9) | 17/648 | 2.6 (1.0–5.7) |
| Human parainfluenzavirus 4 | 1/19 | 5.3 (0.0–33.4) | 5/629 | 0.8 (0.2–2.1) | 6/648 | 0.9 (0.3–2.4) |
| Human bocavirus 1/2/3/4 | 0/19 | 0.0 (0.0–17.6) | 3/629 | 0.5 (0.1–1.6) | 3/648 | 0.5 (0.1–1.6) |
| Human rhinovirus A/B/C | 0/19 | 0.0 (0.0–17.6) | 81/629 | 12.9 (8.9–17.9) | 81/648 | 12.5 (8.6–17.4) |
| Any human coronavirus | 0/19 | 0.0 (0.0–17.6) | 44/629 | 7.0 (4.3–10.6) | 44/648 | 6.8 (4.2–10.3) |
| Human coronavirus 229E | 0/19 | 0.0 (0.0–17.6) | 15/629 | 2.4 (1.0–4.8) | 15/648 | 2.3 (0.9–4.7) |
| Human coronavirus NL63 | 0/19 | 0.0 (0.0–17.6) | 3/629 | 0.5 (0.0–2.1) | 3/648 | 0.5 (0.0–2.1) |
| Human coronavirus OC43 | 0/19 | 0.0 (0.0–17.6) | 26/629 | 4.1 (1.9–7.6) | 26/648 | 4.0 (1.9–7.4) |
| SARS-CoV-2 | 2/19 | 10.5 (1.3–33.1) | 222/629 | 35.3 (21.5–51.1) | 224/648 | 34.6 (21.4–49.7) |

(table continues on next page)

|  | **cRSV-ARI N=136** | | **Non-cRSV-ARI N=2559** | | **Overall N=2695** | |
| --- | --- | --- | --- | --- | --- | --- |
|  | **n/N** | **% (95% CI)** | **n/N** | **% (95% CI)** | **n/N** | **% (95% CI)** |
| **Year 2** |  |  |  |  |  |  |
| Any respiratory viruses | 14/76 | 18.4 (7.5–34.9) | 609/989 | 61.6 (51.5–71.0) | 623/1065 | 58.5 (48.8–67.8) |
| Any influenza A or B virus | 5/76 | 6.6 (2.2–14.7) | 75/989 | 7.6 (4.7–11.4) | 80/1065 | 7.5 (4.8–11.2) |
| Any influenza A subtype | 5/76 | 6.6 (2.2–14.7) | 72/989 | 7.3 (4.4–11.3) | 77/1065 | 7.2 (4.4–11.0) |
| Human influenza A virus | 2/76 | 2.6 (0.3–9.2) | 62/989 | 6.3 (3.6–10.0) | 64/1065 | 6.0 (3.6–9.4) |
| subtype H1pdm09 | 2/76 | 2.6 (0.2–10.0) | 25/989 | 2.5 (1.3–4.5) | 27/1065 | 2.5 (1.3–4.4) |
| subtype H3 | 2/76 | 2.6 (0.3–9.2) | 46/989 | 4.7 (2.9–7.1) | 48/1065 | 4.5 (2.8–6.8) |
| Human influenza B virus | 0/76 | 0.0 (0.0–4.7) | 3/989 | 0.3 (0.1–0.9) | 3/1065 | 0.3 (0.1–0.9) |
| Human adenovirus | 0/76 | 0.0 (0.0–4.7) | 8/989 | 0.8 (0.3–1.6) | 8/1065 | 0.8 (0.3–1.5) |
| Human metapneumovirus | 2/76 | 2.6 (0.3–9.2) | 59/989 | 6.0 (3.7–9.0) | 61/1065 | 5.7 (3.6–8.5) |
| Human enterovirus | 0/76 | 0.0 (0.0–4.7) | 5/989 | 0.5 (0.2–1.2) | 5/1065 | 0.5 (0.2–1.1) |
| Any human parainfluenzavirus | 0/76 | 0.0 (0.0–4.7) | 52/989 | 5.3 (3.7–7.3) | 52/1065 | 4.9 (3.4–6.8) |
| Human parainfluenzavirus 1 | 0/76 | 0.0 (0.0–4.7) | 3/989 | 0.3 (0.1–0.9) | 3/1065 | 0.3 (0.1–0.8) |
| Human parainfluenzavirus 2 | 0/76 | 0.0 (0.0–4.7) | 3/989 | 0.3 (0.0–1.2) | 3/1065 | 0.3 (0.0–1.1) |
| Human parainfluenzavirus 3 | 0/76 | 0.0 (0.0–4.7) | 35/989 | 3.5 (2.3–5.2) | 35/1065 | 3.3 (2.2–4.8) |
| Human parainfluenzavirus 4 | 0/76 | 0.0 (0.0–4.7) | 11/989 | 1.1 (0.6–2.0) | 11/1065 | 1.0 (0.5–1.8) |
| Human bocavirus 1/2/3/4 | 0/76 | 0.0 (0.0–4.7) | 5/989 | 0.5 (0.0–2.3) | 5/1065 | 0.5 (0.0–2.1) |
| Human rhinovirus A/B/C | 4/76 | 5.3 (1.3–13.6) | 183/989 | 18.5 (13.7–24.1) | 187/1065 | 17.6 (12.9–23.1) |
| Any human coronavirus | 0/76 | 0.0 (0.0–4.7) | 55/989 | 5.6 (4.2–7.2) | 55/1065 | 5.2 (3.9–6.7) |
| Human coronavirus 229E | 0/76 | 0.0 (0.0–4.7) | 12/989 | 1.2 (0.6–2.1) | 12/1065 | 1.1 (0.6–2.0) |
| Human coronavirus NL63 | 0/76 | 0.0 (0.0–4.7) | 15/989 | 1.5 (0.9–2.5) | 15/1065 | 1.4 (0.8–2.3) |
| Human coronavirus OC43 | 0/76 | 0.0 (0.0–4.7) | 28/989 | 2.8 (1.7–4.5) | 28/1065 | 2.6 (1.6–4.1) |
| SARS-CoV-2 | 8/76 | 10.5 (4.7–19.7) | 264/989 | 26.7 (22.2–31.5) | 272/1065 | 25.5 (21.2–30.3) |

(table continues on next page)

|  | **cRSV-ARI N=136** | | **Non-cRSV-ARI N=2559** | | **Overall N=2695** | |
| --- | --- | --- | --- | --- | --- | --- |
|  | **n/N** | **% (95% CI)** | **n/N** | **% (95% CI)** | **n/N** | **% (95% CI)** |
| **Season 3** |  |  |  |  |  |  |
| Any respiratory viruses | 7/41 | 17.1 (7.2–32.1) | 504/941 | 53.6 (44.8–62.1) | 511/982 | 52.0 (43.7–60.3) |
| Any influenza A or B virus | 2/41 | 4.9 (0.6–16.5) | 81/941 | 8.6 (6.2–11.6) | 83/982 | 8.5 (6.1–11.3) |
| Any influenza A subtype | 2/41 | 4.9 (0.6–16.5) | 80/941 | 8.5 (6.1–11.5) | 82/982 | 8.4 (6.1–11.2) |
| Human influenza A virus | 1/41 | 2.4 (0.1–12.9) | 68/941 | 7.2 (4.8–10.4) | 69/982 | 7.0 (4.7–10.1) |
| subtype H1pdm09 | 1/41 | 2.4 (0.1–12.9) | 67/941 | 7.1 (4.8–10.1) | 68/982 | 6.9 (4.7–9.8) |
| subtype H3 | 1/41 | 2.4 (0.0–13.5) | 8/941 | 0.9 (0.3–1.9) | 9/982 | 0.9 (0.4–1.8) |
| Human influenza B virus | 0/41 | 0.0 (0.0–8.6) | 1/941 | 0.1 (0.0–0.7) | 1/982 | 0.1 (0.0–0.6) |
| Human adenovirus | 0/41 | 0.0 (0.0–8.6) | 3/941 | 0.3 (0.0–1.1) | 3/982 | 0.3 (0.0–1.1) |
| Human metapneumovirus | 0/41 | 0.0 (0.0–8.6) | 59/941 | 6.3 (4.1–9.1) | 59/982 | 6.0 (3.9–8.8) |
| Human enterovirus | 0/41 | 0.0 (0.0–8.6) | 6/941 | 0.6 (0.2–1.4) | 6/982 | 0.6 (0.2–1.4) |
| Any human parainfluenzavirus | 0/41 | 0.0 (0.0–8.6) | 33/941 | 3.5 (2.4–4.9) | 33/982 | 3.4 (2.3–4.7) |
| Human parainfluenzavirus 1 | 0/41 | 0.0 (0.0–8.6) | 8/941 | 0.9 (0.4–1.7) | 8/982 | 0.8 (0.4–1.6) |
| Human parainfluenzavirus 2 | - | - | - | - | - | - |
| Human parainfluenzavirus 3 | 0/41 | 0.0 (0.0–8.6) | 16/941 | 1.7 (1.0–2.7) | 16/982 | 1.6 (0.9–2.6) |
| Human parainfluenzavirus 4 | 0/41 | 0.0 (0.0–8.6) | 10/941 | 1.1 (0.5–1.9) | 10/982 | 1.0 (0.5–1.9) |
| Human rhinovirus A/B/C | 1/41 | 2.4 (0.0–13.5) | 150/941 | 15.9 (11.6–21.1) | 151/982 | 15.4 (11.2–20.3) |
| Any human coronavirus | 0/41 | 0.0 (0.0–8.6) | 69/941 | 7.3 (4.9–10.4) | 69/982 | 7.0 (4.7–10) |
| Human coronavirus 229E | 0/41 | 0.0 (0.0–8.6) | 11/941 | 1.2 (0.3–2.9) | 11/982 | 1.1 (0.3–2.7) |
| Human coronavirus NL63 | 0/41 | 0.0 (0.0–8.6) | 7/941 | 0.7 (0.3–1.7) | 7/982 | 0.7 (0.2–1.6) |
| Human coronavirus OC43 | 0/41 | 0.0 (0.0–8.6) | 52/941 | 5.5 (3.7–7.8) | 52/982 | 5.3 (3.6–7.5) |
| SARS-CoV-2 | 4/41 | 9.8 (2.7–23.1) | 147/941 | 15.6 (11.5–20.6) | 151/982 | 15.4 (11.3–20.2) |

Year 1, 01 October 2021 to 30 September 2022; Year 2, 01 October 2022 to 30 September 2023; Season 3, 01 October 2023 to 30 April 2024.

ARI, acute respiratory infection; CI, confidence interval; cRSV-ARI, RT-PCR confirmed RSV-ARI; N, number of participants in each group/number of participants with data; n, number of participants with positive RT-PCR test; RSV, respiratory syncytial virus; SARS-CoV-2, severe acute respiratory syndrome coronavirus 2; RT-PCR, reverse transcription polymerase chain reaction.

References

1. Terns Riera M, Prato R, Pérez-Rubio A, et al. Health-related quality of life in European older adults with respiratory syncytial virus over three respiratory syncytial virus seasons. Clin Infect Dis **2025**.
